# Changes in all-cause and cause-specific mortality during the first year of the COVID-19 pandemic in Minnesota: population-based study

**DOI:** 10.1186/s12889-022-14743-z

**Published:** 2022-12-07

**Authors:** Rozalina G. McCoy, Ronna L. Campbell, Aidan F. Mullan, Colin M. Bucks, Casey M. Clements, R. Ross Reichard, Molly M. Jeffery

**Affiliations:** 1 Division of Community Internal Medicine, Geriatrics, and Palliative Care. Department of Medicine, Mayo Clinic, 200 First Street SW, Rochester, MN 55905 USA; 2 Mayo Clinic Robert D. and Patricia E. Kern Center for the Science of Health Care Delivery, Rochester, MN 55905 USA; 3 Mayo Clinic Ambulance, Rochester, MN 55905 USA; 4 Division of Health Care Delivery Research, Mayo Clinic, Rochester, MN 55905 USA; 5 Department of Emergency Medicine, Mayo Clinic, Rochester, MN 55905 USA; 6 Department of Quantitative Health Sciences, Mayo Clinic, Rochester, MN 55905 USA; 7 Department of Laboratory Medicine and Pathology, Mayo Clinic, Rochester, MN 55905 USA

**Keywords:** COVID-19, Mortality, Epidemiology, Pandemic, Population health, Rural

## Abstract

**Background:**

The COVID-19 pandemic resulted in unprecedented increases in mortality in the U.S. and worldwide. To better understand the impact of the COVID-19 pandemic on mortality in the state of Minnesota, U.S.A., we characterize the changes in the causes of death during 2020 (COVID-19 period), compared to 2018–2019 (baseline period), assessing for differences across ages, races, ethnicities, sexes, and geographic characteristics.

**Methods:**

Longitudinal population-based study using Minnesota death certificate data, 2018–2020. Using Poisson regression models adjusted for age and sex, we calculated all-cause and cause-specific (by underlying causes of death) mortality rates per 100,000 Minnesotans, the demographics of the deceased, and years of life lost (YLL) using the Chiang’s life table method in 2020 relative to 2018–2019.

**Results:**

We identified 89,910 deaths in 2018–2019 and 52,030 deaths in 2020. The mean daily mortality rate increased from 123.1 (SD 11.7) in 2018–2019 to 144.2 (SD 22.1) in 2020. COVID-19 comprised 9.9% of deaths in 2020. Other categories of causes of death with significant increases in 2020 compared to 2018–2019 included assault by firearms (RR 1.68, 95% CI 1.34–2.11), accidental poisonings (RR 1.49, 95% CI 1.37–1.61), malnutrition (RR 1.48, 95% CI 1.17–1.87), alcoholic liver disease (RR, 95% CI 1.14–1.40), and cirrhosis and other chronic liver diseases (RR 1.28, 95% CI 1.09–1.50). Mortality rates due to COVID-19 and non-COVID-19 causes were higher among racial and ethnic minority groups, older adults, and non-rural residents.

**Conclusions:**

The COVID-19 pandemic was associated with a 17% increase in the death rate in Minnesota relative to 2018–2019, driven by both COVID-19 and non-COVID-19 causes. As the COVID-19 pandemic enters its third year, it is imperative to examine and address the factors contributing to excess mortality in the short-term and monitor for additional morbidity and mortality in the years to come.

**Supplementary Information:**

The online version contains supplementary material available at 10.1186/s12889-022-14743-z.

## Background

In December 2019, the SARS-CoV-2 virus that causes COVID-19 disease emerged in Wuhan, China. It rapidly spread across the globe and was declared a global pandemic on March 11, 2020 by the World Health Organization. By the end of 2020, COVID-19 was identified as the third leading age-adjusted cause of death in the U.S. [1] During the same period, wide-spread societal responses to the pandemic dramatically changed human interactions and movement as well as the access to and delivery of healthcare. This created the potential for other significant changes to mortality patterns that are not directly attributable to COVID-19 infection. Studies conducted early in the pandemic consistently demonstrated an increase in deaths driven by both COVID-19 infection and other – related and unrelated – causes. However, those studies examined the early months of the pandemic, [2–5] used provisional and not finalized death certificate data, [1–8] focused on singular categories of causes of death, [2, 4, 6] assessed narrow age subgroups, [3] or were conducted in early geographic hotspots of COVID-19 infections [9]. We therefore sought to characterize the changes in the causes of death during the first year of the pandemic compared to the two prior years, assessing for differences across ages, races, ethnicities, sexes, and rurality using finalized population death certificate data for the state of Minnesota, U.S.A.

Studies using provisional data have examined the impact of the COVID-19 pandemic on mortality, [2, 5–7, 10, 11] demonstrating both high rates of mortality secondary to COVID-19 as well as substantial increases in overall mortality and deaths due to heart disease, Alzheimer’s disease, diabetes, and unintentional injury. Other studies revealed disproportionate increases in both COVID-19 and all-cause mortality among racial and ethnic minority populations [3, 8, 9, 12]. However, there is limited granular data about the changes in all-cause and cause-specific mortality on the population level for the full first year of the pandemic and for subgroups of people based on demographics. More granular data could identify populations at increased risk of death from specific etiologies and inform public health interventions both now and in the future.

To address these knowledge gaps, we analyzed all finalized death certificates filed in Minnesota between 2018 and 2019 (pre-pandemic period) and in all of 2020 (first year of the pandemic) to characterize the changes in all-cause and cause-specific mortality, and years of life lost, in the overall Minnesota population as well in subgroups by decedents’ age, sex, race/ethnicity, and geographic (rural versus non-rural) characteristics.

## Methods

### Study design and setting

This is a longitudinal analysis of Minnesota death certificate data, subset into two primary time periods: 2018–2019 (pre-pandemic) and 2020 (pandemic). Study results are reported in accordance with Strengthening the Reporting of Observational Studies in Epidemiology (STROBE) guidelines for observational research studies [13].

### Outcomes

Mortality rates per 100,000 people in Minnesota were calculated relative to annual population counts reported by the U.S. Census Bureau [14] and the Minnesota State Demographic Center [15]. Population counts for specific demographic subgroups were retrieved for 2019 using the IPUMS USA dataset, a publicly available source of American Community Survey microdata [16]. Demographic-specific populations for 2018 and 2020 were estimated using the population proportions for 2019.

We used the underlying cause of death to categorize non-COVID-19 deaths. Any death listing COVID-19 as the underlying cause of death or one of four contributing causes of death was classified as a COVID-19 death. Causes of deaths were identified in the data using International Classification of Diseases, 10th Revision (ICD-10) codes and grouped using the 113 cause-of-death recode, a mutually exclusive and collectively exhaustive categorization of all causes of death [17]. The cause of death is considered finalized after it has undergone coding and official categorization based on ICD-10 codes using an established system of rules. Accidental poisoning deaths were identified using the underlying cause of death, then assessed for substances contributing to the poisoning using all text fields on the death certificate (e.g., all 5 causes of death, other significant factors, how injury occurred, etc.). Substances contributing to death were categorized as opioids, other drugs, and non-drugs (e.g., carbon monoxide, laundry detergent).

### Independent variables

Decedent demographics were taken from the death certificate, including age, sex, race and ethnicity, education level, marital status, place of death, and place of residence. Counties of residency and death were categorized as rural or not rural as defined by the Office of Rural Health Policy, Health Resources and Services Administration [18].

### Statistical analysis

Causes of death during each time period were summarized with frequency counts and mortality rates. Deaths were compared between time periods using rate ratios (RRs) and 95% confidence intervals (CI) calculated using the Miettinen-Nurminen procedure [19]. These analyses were conducted for the entire population and separately for women and men. *P*-values were calculated using Wald’s tests on the log-transform of the RR. To account for the number of comparisons being made, all *p*-values were corrected using the Benjamini-Hochberg correction [20]. Statistical tests were two-sided and corrected *p*-values < 0.05 were considered significant, though with large sample sizes more attention should be paid to effect size than *p*-values.

For each cause of death, demographic-specific rates of death were compared between 2018-2019 and 2020 using Poisson regression models accounting for age and sex, along with an interaction term for time period. An offset term for population per demographic group was included to ensure the Poisson model was measuring demographic-specific death rates. *P*-values are reported for the interaction [21] between the demographic group and time period to determine whether a statistically significant change in death rates occurred for the group between 2018-2019 and 2020. Multivariable adjustment was only applied to the reported adjusted *p*-values; all reported RRs were based on the observed death rates for each demographic group and not adjusted for any other demographics. To ensure that sufficient information was present to test for a change in deaths between time periods, regression models were fit only for demographic groups with at least 5 deaths in both 2018–2019 and 2020.

For deaths due to COVID-19, the time period was not relevant because no COVID-19 deaths occurred during 2018–2019. In this case, Poisson models were fit to compare rates within levels of each demographic variable. Univariable models were fit for each demographic, along with a multivariable model adjusting for age and sex. These models were fit with an offset term for population as above. Statistical significance for each demographic variable was determined using likelihood ratio tests to assess whether death rates due to COVID-19 differed across the levels of a given demographic variable.

To calculate years of life lost (YLL), age- and sex-stratified life expectancies were calculated using the Chiang’s life table method [22] based on our population and mortality data for Minnesota. YLL were calculated for each Minnesota death between 2018 and 2020 as the difference between a person’s age at death and predicted life expectancy given the person’s age and sex. To determine the excess deaths potentially caused by the COVID-19 pandemic, the YLL from 2020 were compared to the expected YLL in 2020 assuming the same rates of death as in 2018–2019 but projected onto the 2020 population. Differences are reported both as excess YLL and the percent change. Additionally, the total YLL from non-COVID-19 causes were compared to the projected YLL for 2020 to quantify the excess YLL brought about by the COVID-19 pandemic but not attributable to the infection itself.

All analyses were conducted using R version 3.6.2.

## Results

We identified a total of 89,910 deaths in Minnesota in 2018–2019 and 52,030 deaths in 2020. Deaths in 2018–2019 totaled 1,003,220 YLL overall or 8920.8 YLL per 100,000 person-years. Using this as the baseline rate for YLL in the population, we would expect 509,064 YLL in Minnesota for 2020. However, we observed 568,881 YLL for Minnesota during this time, an excess of 59,817 YLL and an 11.8% increase over the prior period (*p* < 0.001).

There were significant increases in the death rate per 100,000 persons in all age groups 15 years and older, while mortality among children 4 years and younger decreased (Table 1). Deaths increased most among Hispanic Minnesotans (RR 1.55; 95% CI 1.41–1.70) and least among non-Hispanic White Minnesotans (RR 1.12; 95% CI 1.11–1.14). Increases in death rates were similar among non-rural residents (RR 1.15; 95% CI 1.14–1.17) and rural residents (RR 1.11; 95% CI 1.09–1.13). The mean daily mortality rate increased from 123.1 (SD 11.7) in 2018–2019 to 144.2 (SD 22.1) in 2020 (Wilcoxon test *p* < 0.001). The mean daily rate of non-COVID deaths in 2020 (128.1 [SD 12.1]) was also higher than the total 2018–2019 daily death rate (Wilcoxon test *p* < 0.001). Mortality data stratified by sex are presented in Supplemental Table 1, with similar trends in death rates across the sexes in 2020 as compared to 2018–2019, though men had consistently higher mortality rates than women in both time periods.

**Table 1:** Minnesota All-Cause Mortality, by Demographic Subgroup, 2018–2020

	2018–2019		2020		Rate Ratio			
Group	Deaths Total	Deaths Per 100,000	Deaths Total	Deaths Per 100,000	RR	95% CI	Unadj. ***P***-Value	Adj. ***P***-Value^**1**^
**Age Group**								
0–4 years	786	111.3	228	63.6	0.57	0.49–0.66	< 0.001*	< 0.001*
5–14 years	176	12.0	78	10.5	0.87	0.67–1.14	0.35	0.24
15–24 years	812	57.5	468	65.3	1.14	1.01–1.27	0.03 *	0.05 *
25–34 years	1438	92.1	956	120.7	1.31	1.21–1.42	< 0.001*	< 0.001*
35–44 years	1959	132.4	1251	166.6	1.26	1.17–1.35	< 0.001*	< 0.001*
45–64 years	14,182	502.9	7935	554.5	1.10	1.07–1.13	< 0.001*	< 0.001*
65–84 years	37,012	2337.8	22,033	2743.2	1.17	1.15–1.19	< 0.001*	< 0.001*
≥ 85 years	33,545	15,121.1	18,972	16,859.8	1.11	1.10–1.14	< 0.001*	< 0.001*
**Sex**								
Male	45,350	814.4	26,509	938.1	1.15	1.13–1.17	< 0.001*	< 0.001*
Female	44,558	784.9	25,521	885.9	1.13	1.11–1.15	< 0.001*	< 0.001*
**Race**								
Hispanic	985	152.7	774	236.4	1.55	1.41–1.70	< 0.001*	< 0.001*
Non-Hispanic American Indian	1160	942.1	723	1158.7	1.23	1.12–1.35	< 0.001*	< 0.001*
Non-Hispanic Asian/Pacific Islander	1559	251.8	1059	336.9	1.34	1.24–1.45	< 0.001*	< 0.001*
Non-Hispanic Black	3129	395.3	2096	521.5	1.32	1.25–1.39	< 0.001*	< 0.001*
Non-Hispanic White	82,850	951.7	47,222	1069.1	1.12	1.11–1.14	< 0.001*	< 0.001*
**County of Residency**								
Non-Rural Resident	64,445	733.6	37,727	845.7	1.15	1.14–1.17	< 0.001*	< 0.001*
Rural Resident	25,465	1035.0	14,303	1148.36	1.11	1.09–1.13	< 0.001*	< 0.001*

Shown are the crude (unadjusted) mortality rates and rate ratios (RR) for each demographic subgroup in 2018–2019 and 2020. ^1^ *P*-values adjusted using multivariable Poisson regression accounting for age and sex. *Indicates statistical significance at *p* < 0.05 after correcting for multiple comparisons using the Benjamini-Hochberg correction

COVID-19 was a listed cause of 5155 deaths in 2020, comprising 9.9% of all deaths in 2020 in Minnesota (Table 2). This corresponds to 35,057 YLL from deaths attributed to COVID-19, leaving 533,824 YLL from non-COVID causes (Table 3). When only considering the observed non-COVID YLL in 2020 to those predicted using 2018–2019 estimates, there was an excess of 4.9% or 24,760 additional YLL (*p* < 0.001).

**Table 2:** Minnesota Causes of Death, 2018–2020

	2018–2019 (***N*** = 89,910)		2020 (***N*** = 52,030)		Rate Ratio (RR)		
Cause of Death	Deaths Total	Deaths Per 100,000	Deaths Total	Deaths Per 100,000	RR	95% CI	***P***-Value
Malignant Neoplasms	20,111	178.83	10,040	175.94	0.98	0.96–1.01	0.28
Cardiovascular Diseases	18,983	168.80	9748	170.82	1.01	0.99–1.04	0.44
COVID-19	0	0	5155	90.34	–	–	–
Alzheimer’s Disease	4982	44.30	2551	44.70	1.01	0.96–1.06	0.79
Cerebrovascular Diseases	4584	40.76	2257	39.55	0.97	0.92–1.02	0.35
Chronic Lower Respiratory Diseases	4592	40.83	2179	38.18	0.94	0.89–0.98	0.03 *
Diabetes	2679	23.82	1462	25.62	1.08	1.01–1.15	0.06
Accidental Poisoning or Exposure to Noxious Substances	1313	11.68	990	17.35	1.49	1.37–1.61	< 0.001 *
Infectious Diseases	1542	13.71	837	14.67	1.07	0.98–1.16	0.23
Kidney Disease	1667	14.82	828	14.51	0.98	0.90–1.06	0.72
Parkinson’s Disease	1447	12.87	782	13.70	1.07	0.98–1.16	0.27
Suicide	1514	13.46	717	12.56	0.93	0.85–1.02	0.24
Alcoholic Liver Disease	963	8.56	617	10.81	1.26	1.14–1.40	< 0.001 *
Motor Vehicle Accident	866	7.70	466	8.17	1.06	0.95–1.19	0.43
Pneumonia/Other Acute Lower Respiratory Infections	879	7.82	424	7.43	0.95	0.85–1.07	0.49
Other Unintentional Injury	452	4.02	266	4.66	1.16	1.00–1.35	0.13
Cirrhosis/Other Chronic Liver Disease	402	3.57	261	4.57	1.28	1.09–1.50	0.006 *
Assault by Discharge of Firearms	163	1.45	139	2.44	1.68	1.34–2.11	< 0.001 *
Malnutrition	164	1.46	123	2.16	1.48	1.17–1.87	0.004 *
Influenza	322	2.86	109	1.91	0.67	0.54–0.83	0.001 *
Assault by Other Means	99	0.88	51	0.89	1.02	0.72–1.42	1.00
Legal Intervention	24	0.21	12	0.21	0.99	0.49–1.97	1.00
All Others	22,162	197.07	12,016	210.57	1.07	1.05–1.09	< 0.001 *

Shown are the crude (unadjusted) mortality rates for each demographic subgroup in 2018–2019 and 2020. Deaths are categorized based on the primary cause as reported on the death certificate, with the exception of COVID-19 deaths that were categorized as such if the primary or one of the first four contributing causes of death was classified as a COVID-19 death. *Indicates statistical significance at *p* < 0.05 after correcting for multiple comparisons using the Benjamini-Hochberg correction

**Table 3:** Minnesota Years of Life Lost by Cause of Death, 2018–2020

	2018–2019 (***N*** = 89,910)			2020 (***N*** = 52,030)			
Cause of Death	Deaths Total	YLL	YLL Per 100,000	Deaths Total	YLL	YLL Per 100,000	Percent Change
Malignant Neoplasms	20,111	261,359	2324.0	10,040	129,398	2267.5	−2.5%
Cardiovascular Diseases	18,983	145,411	1293.0	9748	77,055	1350.3	+ 4.4%
Suicide	1514	55,930	497.3	717	25,239	442.3	−11.1%
Accidental Poisoning or Exposure to Noxious Substances	1313	51,177	455.1	990	39,983	700.7	+ 54.0%
Chronic Lower Respiratory Diseases	4592	40,430	359.5	2179	20,333	356.3	−0.9%
Diabetes	2679	32,384	288.0	1462	17,949	314.5	+ 9.2%
Cerebrovascular Diseases	4584	31,540	280.5	2257	15,639	274.1	−2.3%
Motor Vehicle Accident	866	30,257	269.1	466	16,536	289.8	+ 7.7%
Alcoholic Liver Disease	963	24,881	221.2	617	16,675	292.2	+ 32.1%
Infectious Diseases	1542	19,924	177.2	837	10,574	185.3	+ 4.6%
Kidney Disease	1667	12,492	111.1	828	6457	113.2	+ 1.9%
Alzheimer’s Disease	4982	12,123	107.8	2551	5514	96.6	−10.4%
Other Unintentional Injury	452	8504	75.6	266	4627	81.1	+ 7.3%
Assault by Discharge of Firearms	163	7915	70.4	139	6748	118.2	+ 67.9%
Pneumonia/Other Acute Lower Respiratory Infections	879	7796	69.3	424	3330	58.4	−15.7%
Parkinson’s Disease	1447	7266	64.6	782	4226	74.1	+ 14.7%
Cirrhosis/Other Chronic Liver Disease	402	6777	60.3	261	4730	82.9	+ 37.5%
Assault by Other Means	99	4630	41.2	51	2386	41.8	+ 1.5%
Influenza	322	3122	27.8	109	1742	30.5	+ 9.7%
Malnutrition	164	1168	10.4	123	757	13.3	+ 27.9%
Legal Intervention	24	1068	9.5	12	582	10.2	+ 7.4%
COVID-19	0	0	0	5155	35,057	614.3	–
All Others	22,162	237,064	2108.0	12,016	123,344	2161.5	+ 2.5%

Other categories of causes of death with significant increases in 2020 compared to 2018–2019 included assault by firearms (RR 1.68, 95% CI 1.34–2.11), accidental poisonings (RR 1.49, 95% CI 1.37–1.61), alcoholic liver disease (RR, 95% CI 1.14–1.40), cirrhosis and other chronic liver diseases (RR 1.28, 95% CI 1.09–1.50), and malnutrition (RR 1.48, 95% CI 1.17–1.87) (Table 2). In contrast, deaths related to other chronic lower respiratory diseases (RR 0.94, 95% CI 0.89–0.98) and influenza diseases (RR 0.67, 95% CI 0.54–0.83) decreased in 2020. The corresponding YLL due to each of these causes of death are shown in Table 3. There were no significant changes in the other causes of death, including deaths from cancer, cardiovascular disease, cerebrovascular disease, dementia, kidney disease, motor vehicle collisions, or suicide, as outlined in Table 2.

### Cause-specific mortality rates and demographics

The highest mortality rate for COVID-19 was among Minnesotans aged ≥85 years (2072.4 per 100,000), American Indians (105.8 per 100,000), rural residents (97.1 per 100,000), and men (96.1 per 100,000) (Table 4). Deaths due to assault by firearms were increased in individuals 15–34 and 45–64 years of age, with the greatest increase among those 45–64 years (RR 2.30, 95% CI 1.23–4.32) and women (RR 2.28, 95% CI 1.24–4.22) (Supplemental Table 2). There were also significant increases in deaths due to assault by firearms among Black Minnesotans (RR 1.82, 95% CI 1.35–2.45), non-rural residents (RR 1.73, 95% CI 1.35–2.20), and men (RR 1.60, 95% CI 1.25–2.04). When firearms-related deaths were stratified by sex (Supplemental Table 3), we found that the majority of these deaths were among men, though women saw a larger increase in firearms-related mortality in 2020 relative to 2018–2019.

**Table 4:** Minnesota Deaths from COVID-19, by Demographic Subgroup, 2020

Group	Deaths Total	Deaths Per 100,000	Unadj. ***P***-Value^**1**^	Adj. ***P***-Value^**2**^
**Age Group**			< 0.001 *	< 0.001 *
0–4 years	0	0.0		
5–14 years	0	0.0		
15–24 years	3	0.4		
25–34 years	14	1.8		
35–44 years	40	5.3		
45–64 years	486	34.0		
65–84 years	2279	283.7		
≥ 85 years	2332	2072.4		
**Sex**			< 0.001 *	< 0.001 *
Male	2715	96.1		
Female	2440	84.7		
**Race**			< 0.001 *	< 0.001 *
Hispanic	155	47.4		
Non-Hispanic American Indian	66	105.8		
Non-Hispanic Asian/Pacific Islander	195	62.0		
Non-Hispanic Black	258	64.2		
Non-Hispanic White	4464	101.1		
**County of Residency**			< 0.001 *	< 0.001 *
Non-Rural Resident	3946	88.5		
Rural Resident	1209	97.1		

Shown are the crude (unadjusted) mortality rates for each demographic subgroup in 2020. ^1^ *P*-value determined from univariable Poisson regression. ^2^ *P*-value determined from multivariable Poisson regression accounting for age and sex. *Indicates statistical significance at *p* < 0.05 after correcting for multiple comparisons using the Benjamini-Hochberg correction

Deaths due to accidental poisoning/overdose increased 49% in 2020 relative to 2018–19 (RR 1.49, 95% CI 1.37–1.61). Although the category includes all types of poisonings, nearly all accidental poisonings included one or more drug (98.4% across the 3 years). The proportion of accidental poisoning deaths that included one or more opioids increased from 55.6% in 2018–2019 to 63.3% in 2020 (RR 1.69, 95% CI 1.52–1.88). Accidental poisoning deaths increased substantially in nearly all demographic groups (Supplemental Table 4). The greatest increases occurred among racial and ethnic minority populations: non-Hispanic Black Minnesotans (RR 1.72, 95% CI 1.41–2.11), Hispanic Minnesotans (RR 1.71, 95% CI 1.14–2.54), and non-Hispanic American Indian Minnesotans (RR 1.68, 95% CI 1.29–2.20). People aged 15–64 experienced increased mortality from accidental poisoning, with the greatest increases in those aged 15–24 (RR 1.64, 95% CI 1.28–2.09) and 25–34 (RR 1.64, 95% CI 1.40–1.92). Sex-stratified analyses (Supplemental Table 5) revealed that men had higher rates of mortality from accidental poisoning than women in both time periods, though both sexes saw similar increases in 2020 relative to 2018–2019.

Deaths due to malnutrition were increased among residents aged 85 years and older (RR 1.76, 95% CI 1.27–2.45), women (RR 1.64, 95% CI 1.23–2.19), White individuals (RR 1.44, 95% CI 1.13–1.83) and rural residents (RR 2.50, 95% CI 1.55–4.04) (Supplemental Table 6). Their rates of death in 2020 were 60.4, 2.95, 2.6, and 3.1 per 100,000, respectively. Sex-stratified analyses (Supplemental Table 7) revealed that older (those aged ≥85 years) and White women had higher rates of mortality from malnutrition than similarly aged and White men in both time periods, and that the COVID-19-period increase in malnutrition-related deaths was limited primarily to White women ≥85 years old living in rural areas.

Deaths due to alcoholic liver disease increased among residents aged 25–44 years and ≥ 85 years. The largest increases occurred among individuals aged 85 years and older (RR 15.77, 95% CI 1.97–126.10), men (RR 1.19, 95% CI 1.04–1.35), White individuals (RR 1.26, 95% CI 1.13–1.40), and non-rural residents (RR 1.29, 95% CI 1.13–1.44) (Supplemental Table 8). Sex-stratified analyses (Supplemental Table 9) found these deaths to be more frequent among men, with the exception of American Indian women, whose death rates due to alcoholic liver disease were higher than among men and any other racial or ethnic group. Increases in deaths due to alcoholic liver disease seen in 2020 relative to 2018–2019, however, were of the same or greater magnitude among women compared to men. Deaths due to cirrhosis and other chronic liver diseases increased the most among those aged 65–84 (RR 1.32, 95% CI 1.07–1.64), non-rural residents (RR 1.29, 95% CI 1.08–1.55), men (RR 1.40, 95% CI 1.14–1.73), and White residents (RR 1.31, 95% CI 1.11–1.55) (Supplemental Table 10). Sex-stratified analyses of deaths related to cirrhosis and other chronic liver disease are shown in Supplemental Table 11; however, due to relatively low event rates, increases in these deaths reached statistical significance only among men aged 65–84 years, White men, and rural residents.

## Discussion

Using complete death certificate data for the state of Minnesota between 2018 and 2020, we found significant increases in death rates in all adult (≥25 years) age groups in 2020 compared to 2018–2019. COVID-19 was a contributing cause of death for nearly 10% of all deaths in 2020, and there were also significant increases in non-COVID-19 deaths due to assault by firearms, accidental poisonings, malnutrition, alcoholic liver disease, as well as cirrhosis and other chronic liver disease. These data underscore the importance of examining finalized death data that has been officially and systematically coded and categorized, as preliminary estimates published early during the pandemic found few excess deaths in Minnesota and nearly all (97%) of these excess deaths were attributed to COVID-19 [11]. We did not find statistically significant increases in deaths due to other causes, including those hypothesized to be affected by pandemic-related changes in daily life and access to health care including cancer, cardiovascular disease, cerebrovascular disease, dementia, kidney disease, motor vehicle collisions, and suicide.

Substantial increases in accidental poisoning/overdose deaths seen in Minnesota mirror national trends identified in other studies during COVID-19 [23]. Emerging evidence suggests multiple potential drivers of this change, including economic stress and financial uncertainty, anxiety about a new infection affecting large segments of society, changes in the illicit drug supply observed at the national level [24] and described by people who use drugs, [25] as well as interruptions in access to mental health and substance disorder treatment [26]. The largest increases in accidental poisoning/overdose deaths in Minnesota were seen among the groups experiencing the highest burden of overdose deaths before the pandemic, including racial and ethnic minority populations, young and middle-aged adults, men, and residents in non-rural areas. A recent analysis of national opioid overdose mortality trends found that Minnesota has the largest disparity in opioid overdose death rates between American Indian and non-Hispanic White people among the ten states where such rates could be calculated [27]. Addressing this crisis must be a public health priority, including supporting the tribal nations’ work to address health, economic, and community well-being [28, 29]. Reducing overdose deaths requires a broad public health approach that addresses both the factors that lead to initiating drug use and the factors that increase the harms associated with it, including increasing access to treatment and harm reduction services and reforming the criminal legal system’s approach to people who use drugs [30].

Prior to COVID-19, alcohol use contributed to 95,000 deaths annually in the U.S., [31] with 5.3% of Americans 12 years and older having alcohol use disorder [32]. Alcohol purchasing and consumption increased substantially in 2020, [33, 34] as the nation faced the stresses and uncertainties related to the pandemic, job loss, social isolation, and social unrest. We therefore were not surprised to find that the rate of deaths from alcoholic liver disease increased 26.3% from 2018 to 2019 to 2020. Deaths from other chronic liver disease similarly increased by 28%, though the data are limited by our inability to precisely delineate the difference between these two categories of causes of death. Deaths increased significantly among all adults ≥25 years but were most pronounced among individuals ≥85 years and women, suggesting a need for increased vigilance and screening for alcohol use disorder and its complications in these populations, particularly during times of isolation and stress. Most of these excess deaths likely represent decompensation of pre-existing chronic alcohol use and associated liver disease, either due to continued or increased alcohol use, lack of treatment, or as a contributing factor to more severe COVID-19 illness or liver disease progression after COVID-19 illness [35, 36]. This early spike in deaths due to liver disease may presage long-term effects of increasing alcohol use and alcohol use disorder within the population. We anticipate that excess deaths due to chronic liver disease, heart disease, and cancer will continue to become apparent over the years to come.

We found that malnutrition deaths increased among the elderly, and in White individuals, women, and rural residents resulting in a 28% increase in YLL due to malnutrition in 2020 relative to 2018–2019. Sex-stratified analyses suggested that increases in malnutrition-related deaths were seen primarily in women 85 years and older, White women, and rural residents. To our knowledge, this has not been previously reported, though our findings are limited by the lack of more explicit categorization and etiologic information. Malnutrition has been shown to be related to environmental and social factors, and vulnerability to malnutrition increases with age. Depression and loneliness [37] are important risk factors for malnutrition among older adults. It is likely that increased social isolation due to the COVID-19 pandemic resulted in exacerbation of underlying risk factors for malnutrition, including loneliness and depression. Residents in rural areas may have been particularly vulnerable to isolation based on decreased proximity to neighbors and smaller communities. Older and White women are also more likely to live alone, [38, 39] underscoring the impact of isolation and lack of economic, logistical, and social support on self-care and sustenance.

Finally, we found increased deaths due to assault discharge of firearms, though not by suicide, and no changes in the fatality rate by motor vehicle accidents. This is consistent with early data available through August 2020 finding an increase in homicides and unintentional injuries; no change in motor vehicle accidents; and a decrease in suicidality [4]. Deaths by discharge of firearms increased by 2.3-fold among individuals 45–64 years old; 1.7-fold among non-rural residents (with no significant increase among rural residents); and 2.6-fold among Hispanic, 1.8-fold among non-Hispanic Black, and 1.6-fold among non-Hispanic White individuals. These deaths mirror those attributed to other “deaths of despair,” [40] including accidental poisoning/overdose and alcoholic liver disease, underscoring the impact of the pandemic, the social justice movement, as well as economic and other stressors on preventable deaths and injuries. The lack of increase in deaths by suicide is consistent with prior studies but is surprising given the increased stress experienced during the pandemic and will need to be further examined.

Importantly, we did not find significant increases in deaths due to many health conditions for which care may have been delayed or deferred as the result of COVID-19 restrictions on elective care as well as patients’ potential hesitancy to seek preventive care. These conditions include cancer, cardiovascular disease, cerebrovascular disease, dementia, and kidney disease. Prior studies [2, 5–7, 10, 11] found mixed results with respect to the impact of the COVID-19 pandemic on these deaths but were limited to the early stages of the pandemic with incomplete ascertainment of deaths and their causes. This lack of increase in mortality secondary to chronic disease is reassuring given evidence that hospitalizations for the management of these conditions decreased, particularly early in the pandemic [41–43]. The reduction in hospitalizations raised concerns about delayed care and later impacts on mortality that are not seen here. Nevertheless, it is possible that long-term sequelae of both delayed/deferred care and COVID-19 infection will result in increased mortality from chronic disease in future years, warranting close surveillance of mortality trends for years and decades to come.

Consistent with prior studies from early in the pandemic, [8, 44, 45] mortality rates due to COVID-19 and non-COVID-19 causes were higher among racial and ethnic minority groups, particularly among American Indians, compared to non-Hispanic White Minnesotans. These findings underscore the pervasive impacts of structural racism on individual and population health [46, 47]. Members of racial and ethnic minority groups are more likely to be employed in fields not amenable to remote work and with higher risk of COVID-19 exposure, [46, 48] live in multigenerational households, and have less access to timely medical care [46, 49–51]. American Indian communities living in reservations have long experienced inadequate access to medical care secondary to underfunded and underresourced healthcare facilities [52, 53]. It is therefore critical not only to escalate efforts to address the structural causes of heightened infection risk and adverse health outcomes among racial and ethnic minority groups, but to also mitigate the effects of higher morbidity experienced by minority populations during the pandemic. This includes developing tailored, culturally appropriate, and accessible care models to support people experiencing long-term sequela of COVID-19 infection as well as people with deteriorating chronic health conditions as the result of delayed care [54].

While this is the first analysis of complete population level data on COVID-19, all-cause, and cause-specific mortality in 2020, this study has important limitations. Our data are limited to residents of Minnesota and may not generalize to other populations. We are not able to directly compare the observed excess deaths in Minnesota to those of other states, as no finalized data for other states or the U.S. overall are available. Provisional estimates published early in the pandemic, however, found a much higher proportion of deaths to be attributed directly to COVID-19, [3, 5, 10, 11] underscoring the delayed effects of COVID-19, the public health measures used to contain in, and decreased access to or utilization of healthcare services for acute and chronic health needs. As with all analyses of death certificates, there is risk for misclassification of the causes of death, including missed cases of COVID-19 and its complications.

## Conclusions

The COVID-19 pandemic was associated with a 17% increase in the death rate in Minnesota relative to 2018–2019, disproportionately affecting racial and ethnic minority populations and older adults. Deaths were driven by COVID-19 and non-COVID-19 causes, with most striking impacts on preventable deaths of despair caused by overdose, alcohol use, and malnutrition. As the COVID-19 pandemic enters its third year, it is imperative to examine and address the factors contributing to excess mortality in the short-term and monitor for additional morbidity and mortality in the years to come.

## Supplementary Information

**Additional file 1: Supplemental Table 1.** Minnesota All-Cause Mortality, by Demographic Subgroup, Stratified by Sex, 2018-2020. **Supplemental Table 2.** Minnesota Deaths by Discharge of Firearms, by Demographic Subgroup, 2018-2020. **Supplemental Table 3.** Minnesota Deaths by Discharge of Firearms, by Demographic Subgroup, Stratified by Sex, 2018-2020. **Supplemental Table 4.** Minnesota Deaths by Accidental Poisoning, by Demographic Subgroup, 2018-2020. **Supplemental Table 5.** Minnesota Deaths by Accidental Poisoning, by Demographic Subgroup, Stratified by Sex, 2018-2020. **Supplemental Table 6.** Minnesota Deaths by Malnutrition, by Demographic Subgroup, 2018-2020. **Supplemental Table 7.** Minnesota Deaths by Malnutrition, by Demographic Subgroup, Stratified by Sex, 2018-2020. **Supplemental Table 8.** Minnesota Deaths from Alcoholic Liver Disease, by Demographic Subgroup, 2018-2020. **Supplemental Table 9.** Minnesota Deaths from Alcoholic Liver Disease, by Demographic Subgroup, Stratified by Sex, 2018-2020. **Supplemental Table 10.** Minnesota Deaths from Cirrhosis or Other Chronic Liver Disease, by Demographic Subgroup, 2018-2020. **Supplemental Table 11.** Minnesota Deaths from Cirrhosis or Other Chronic Liver Disease, by Demographic Subgroup, Stratified by Sex, 2018-2020.

## Data Availability

The data that support the findings of this study are available from Rochester Epidemiology Project restrictions apply to the availability of these data, which were used under license for the current study, and so are not publicly available. Data are however available from the authors upon reasonable request and with permission of Rochester Epidemiology Project at Mayo Clinic at info@rochesterproject.org.

## Abbreviations

CI: Confidence interval
RR: Rate ratio

## References

[1] Ahmad FB, Cisewski JA, Minino A, Anderson RN (2021). Provisional mortality data - United States, 2020. MMWR Morb Mortal Wkly Rep.

[2] Wadhera RK, Shen C, Gondi S, Chen S, Kazi DS, Yeh RW (2021). Cardiovascular deaths during the COVID-19 pandemic in the United States. J Am Coll Cardiol.

[3] Faust JS, Krumholz HM, Du C, Mayes KD, Lin Z, Gilman C (2021). All-cause excess mortality and COVID-19-related mortality among US adults aged 25-44 years, march-July 2020. JAMA.

[4] Faust JS, Du C, Mayes KD, Li S-X, Lin Z, Barnett ML (2021). Mortality from drug overdoses, homicides, unintentional injuries, motor vehicle crashes, and suicides during the pandemic, march-august 2020. JAMA.

[5] Woolf SH, Chapman DA, Sabo RT, Weinberger DM, Hill L, Taylor DDH (2020). Excess deaths from COVID-19 and other causes, march-July 2020. JAMA.

[6] Ran J, Zhao S, Han L, Ge Y, Chong MKC, Cao W (2021). Increase in diabetes mortality associated with COVID-19 pandemic in the U.S. Diabetes Care.

[7] Ahmad FB, Anderson RN (2021). The leading causes of death in the US for 2020. JAMA.

[8] Rossen LM, Branum AM, Ahmad FB, Sutton P, Anderson RN (2020). Excess deaths associated with COVID-19, by Age and race and Ethnicity - United States, January 26-October 3, 2020. MMWR Morb Mortal Wkly Rep.

[9] Simon P, Ho A, Shah MD, Shetgiri R (2021). Trends in mortality from COVID-19 and other leading causes of death among Latino vs white individuals in Los Angeles County, 2011-2020. JAMA.

[10] Weinberger DM, Chen J, Cohen T, Crawford FW, Mostashari F, Olson D (2020). Estimation of excess deaths associated with the COVID-19 pandemic in the United States, march to may 2020. JAMA Intern Med.

[11] Woolf SH, Chapman DA, Sabo RT, Weinberger DM, Hill L (2020). Excess deaths from COVID-19 and other causes, march-April 2020. JAMA.

[12] Acosta AM, Garg S, Pham H, Whitaker M, Anglin O, O'Halloran A (2021). Racial and ethnic disparities in rates of COVID-19-associated hospitalization, intensive care unit admission, and in-hospital death in the United States from march 2020 to February 2021. JAMA Netw Open.

[13] von Elm E, Altman DG, Egger M, Pocock SJ, Gøtzsche PC, Vandenbroucke JP (2007). The strengthening the reporting of observational studies in epidemiology (STROBE) statement: guidelines for reporting observational studies. Ann Intern Med.

[14] U.S. Census Bureau (2021). State Population by Characteristics: 2010-2019.

[15] Minnesota State Demographic Center. Age, Race, & Ethnicity. State population by Age, sex, race, and Hispanic origin 2010–2019 St. Paul: Minnesota State Demographic Center; Cited 2022 March 18. Available from: https://mn.gov/admin/demography/data-by-topic/age-race-ethnicity/.

[16] IPUMS USA U.S (2022). Census data for social, economic, and Health Research: Minnesota population Center.

[17] Heron M. Deaths: Leading Causes for 2017. Natl Vital Stat Rep. 2019;68(6):1–77. 32501203

[18] List of Rural Counties and Designated Eligible Census Tracts in Metropolitan Counties (2018). Office of Rural Health Policy. Health Resources and Services Administration.

[19] Miettinen O, Nurminen M (1985). Comparative analysis of two rates. Stat Med.

[20] Benjamini Y, Hochberg Y (1995). Controlling the false discovery rate: a practical and powerful approach to multiple testing. J R Stat Soc Ser B Methodol.

[21] Frome EL, Checkoway H (1985). Epidemiologic programs for computers and calculators. Use of Poisson regression models in estimating incidence rates and ratios. Am J Epidemiol.

[22] Chiang CL, Organization WH (1979). Life table and mortality analysis.

[23] Friedman JR, Hansen H. Evaluation of increases in drug overdose mortality rates in the US by race and Ethnicity before and during the COVID-19 pandemic. JAMA Psychiatry. 2022;79(4):379–81. 10.1001/jamapsychiatry.2022.0004 PMC8892360 35234815

[24] CDC (2020). Increase in fatal drug overdoses across the United States driven by synthetic opioids before and during the COVID-19 pandemic: Center for Preparedness and Response. Center for Disease Control and Prevention.

[25] Bolinski RS, Walters S, Salisbury-Afshar E, Ouellet LJ, Jenkins WD, Almirol E, et al. The impact of the COVID-19 pandemic on drug use behaviors, fentanyl exposure, and harm reduction service support among people who use drugs in rural settings. Int J Environ Res Public Health. 2022;19(4):2230. 10.3390/ijerph19042230 PMC8872091 35206421

[26] Huskamp HA, Busch AB, Uscher-Pines L, Barnett ML, Riedel L, Mehrotra A (2020). Treatment of opioid use disorder among commercially insured patients in the context of the COVID-19 pandemic. JAMA.

[27] Humphreys K, Shover CL, Andrews CM, Bohnert ASB, Brandeau ML, Caulkins JP (2022). Responding to the opioid crisis in North America and beyond: recommendations of the Stanford-lancet commission. Lancet.

[28] Greenfield B, Russell E, Youngdeer H, Walls M, Alexander C (2019). Reducing Opioid Overdose Deaths in Minnesota: Insights from One Tribal Nation. National Drug Early Warning System (NDEWS) Minnesota HotSpot Study.

[29] Magarati M, Aviles R, Lucero D, S. S, Egashira L, Brown C, et al. Models of tribal promising practices: tribal opioid overdose prevention, care coordination, and data systems. Seattle: Seven Directions, Center for Indigenous Public Health, University of Washington; 2020. Cited March 18, 2022. Available from: https://assets.website-files.com/5d4b3177c03a6439be501a14/5fa99e7d61af7a013709a695_NNPHI-Seven-Directions-Report-100320-v8.pdf.

[30] Park JN, Rouhani S, Beletsky L, Vincent L, Saloner B, Sherman SG (2020). Situating the continuum of overdose risk in the social determinants of health: a new conceptual framework. Milbank Quarterly.

[31] CDC (2020). Alcohol and Public Health: Alcohol-Related Disease Impact (ARDI) Application Atlanta, GA: Centers for Disease Control and Prevention.

[32] SAMHSA (2019). National Survey on drug use and health Rockville, MD: Substance Abuse and Mental Health Services Administration. Center for Behavioral Health Statistics and Quality.

[33] Pollard MS, Tucker JS, Green HD (2020). Changes in adult alcohol use and consequences during the COVID-19 pandemic in the US. JAMA Netw Open.

[34] Alcohol Sales During the COVID-19 Pandemic: Alcohol Epidemiologic Data System. Division of Epidemiology and Prevention Research. National Institute on Alcohol Abuse and Alcoholism. U.S. Department of Health & Human Services.; 2022. updated January 25, 2022; Cited 2022 March 18. Available from: https://pubs.niaaa.nih.gov/publications/surveillance-covid-19/COVSALES.htm.

[35] Singh S, Khan A (2020). Clinical characteristics and outcomes of coronavirus disease 2019 among patients with preexisting liver disease in the United States: a multicenter research network study. Gastroenterology.

[36] Ge J, Pletcher MJ, Lai JC (2021). Outcomes of SARS-CoV-2 infection in patients with chronic liver disease and cirrhosis: a national COVID cohort collaborative study. Gastroenterology.

[37] Eskelinen K, Hartikainen S, Nykänen I (2016). Is loneliness associated with malnutrition in older people?. Int J Gerontol.

[38] Housing America’s Older Adults 2019 Cambridge, MA, USA: Joint Center for Housing Studies of Harvard University; 2019. Cited 2022 Nov 7. Available from: https://www.jchs.harvard.edu/housing-americas-older-adults-2019.

[39] Stepler R (2016). Pew research Center: smaller share of women ages 65 and older are living alone.

[40] Case A, Deaton A (2015). Rising morbidity and mortality in midlife among white non-Hispanic Americans in the 21st century. Proc Natl Acad Sci.

[41] Baum A, Schwartz MD (2020). Admissions to veterans affairs hospitals for emergency conditions during the COVID-19 pandemic. JAMA.

[42] Solomon MD, McNulty EJ, Rana JS, Leong TK, Lee C, Sung SH (2020). The covid-19 pandemic and the incidence of acute myocardial infarction. N Engl J Med.

[43] Bhatt AS, Moscone A, McElrath EE, Varshney AS, Claggett BL, Bhatt DL (2020). Fewer hospitalizations for acute cardiovascular conditions during the COVID-19 pandemic. J Am Coll Cardiol.

[44] Shiels MS, Haque AT, Haozous EA, Albert PS, Almeida JS, García-Closas M, et al. Racial and Ethnic Disparities in Excess Deaths During the COVID-19 Pandemic, March to December 2020. Ann Intern Med. 2021;174(12):1693–99. 10.7326/M21-2134 PMC8489677 34606321

[45] Mackey K, Ayers CK, Kondo KK, Saha S, Advani SM, Young S (2021). Racial and ethnic disparities in COVID-19-related infections, hospitalizations, and deaths : a systematic review. Ann Intern Med.

[46] Zalla LC, Martin CL, Edwards JK, Gartner DR, Noppert GA (2021). A geography of risk: structural racism and coronavirus disease 2019 mortality in the United States. Am J Epidemiol.

[47] Holden TM, Simon MA, Arnold DT, Halloway V, Gerardin J (2022). Structural racism and COVID-19 response: higher risk of exposure drives disparate COVID-19 deaths among black and Hispanic/Latinx residents of Illinois, USA. BMC Public Health.

[48] McClure ES, Vasudevan P, Bailey Z, Patel S, Robinson WR (2020). Racial capitalism within public health—how occupational settings drive COVID-19 disparities. Am J Epidemiol.

[49] Webb Hooper M, Nápoles AM, Pérez-Stable EJ (2020). COVID-19 and racial/ethnic disparities. JAMA.

[50] Lopez L, Hart LH, Katz MH (2021). Racial and ethnic health disparities related to COVID-19. JAMA.

[51] Wiltz JL, Feehan AK, Molinari NM, Ladva CN, Truman BI, Hall J (2022). Racial and ethnic disparities in receipt of medications for treatment of COVID-19 - United States, march 2020-august 2021. MMWR Morb Mortal Wkly Rep.

[52] Graves JM, Mackelprang JL, Amiri S, Abshire DA (2021). Barriers to telemedicine implementation in southwest tribal communities during COVID-19. J Rural Health.

[53] Rodriguez-Lonebear D, Barceló NE, Akee R, Carroll SR. American Indian reservations and COVID-19: correlates of early infection rates in the pandemic. J Public Health Manag Pract. 2020;26(4):371–77. 10.1097/PHH.0000000000001206 PMC7249493 32433389

[54] Jiang DH, McCoy RG (2020). Planning for the post-COVID syndrome: how payers can mitigate long-term complications of the pandemic. J Gen Intern Med.

