# Supplementary material for: Changes in all-cause and cause-specific mortality during the first year of the COVID-19 pandemic in Minnesota: population-based study
Source: BMC Public Health. 2022 Dec 7;22:2291. doi: 10.1186/s12889-022-14743-z (PMC9727873; doi:10.1186/s12889-022-14743-z)
Supplement: Supplementary file 1 — Additional file 1: Supplemental Table 1. Minnesota All-Cause Mortality, by Demographic Subgroup, Stratified by Sex, 2018-2020. Supplemental Table 2. Minnesota Deaths by Discharge of Firearms, by Demographic Subgroup, 2018-2020. Supplemental Table 3. Minnesota Deaths by Discharge of Firearms, by Demographic Subgroup, Stratified by Sex, 2018-2020. Supplemental Table 4. Minnesota Deaths by Accidental Poisoning, by Demographic Subgroup, 2018-2020. Supplemental Table 5. Minnesota Deaths by Accidental Poisoning, by Demographic Subgroup, Stratified by Sex, 2018-2020. Supplemental Table 6. Minnesota Deaths by Malnutrition, by Demographic Subgroup, 2018-2020. Supplemental Table 7. Minnesota Deaths by Malnutrition, by Demographic Subgroup, Stratified by Sex, 2018-2020. Supplemental Table 8. Minnesota Deaths from Alcoholic Liver Disease, by Demographic Subgroup, 2018-2020. Supplemental Table 9. Minnesota Deaths from Alcoholic Liver Disease, by Demographic Subgroup, Stratified by Sex, 2018-2020. Supplemental Table 10. Minnesota Deaths from Cirrhosis or Other Chronic Liver Disease, by Demographic Subgroup, 2018-2020. Supplemental Table 11. Minnesota Deaths from Cirrhosis or Other Chronic Liver Disease, by Demographic Subgroup, Stratified by Sex, 2018-2020. [file 12889_2022_14743_MOESM1_ESM.docx]

**Supplemental Table 1: Minnesota All-Cause Mortality, by Demographic Subgroup, Stratified by Sex, 2018-2020.** Shown are the crude (unadjusted) mortality rates and rate ratios (RR) for each demographic subgroup in 2018-2019 and 2020. *Indicates statistical significance at p<0.05 after correcting for multiple comparisons using the Benjamini-Hochberg correction.

|  | **Females** | | | | **Males** | | | |
| --- | --- | --- | --- | --- | --- | --- | --- | --- |
|  | **Deaths per 100,000** | | **RR**  **(95% CI)** | **P-Value** | **Deaths per 100,000** | | **RR**  **(95% CI)** | **P-Value** |
|  | **2018-2019** | **2020** |  |  | **2018-2019** | **2020** |  |  |
| **Age Group** |  |  |  |  |  |  |  |  |
| 0-4 years | 97.4 | 51.4 | 0.53 (0.42 - 0.66) | <0.001* | 124.3 | 75.6 | 0.61 (0.50 – 0.74) | <0.001* |
| 5-14 years | 10.2 | 6.8 | 0.67 (0.42 – 1.05) | 0.20 | 13.9 | 14.2 | 1.02 (0.73 – 1.43) | >0.99 |
| 15-24 years | 31.5 | 36.4 | 1.16 (0.93 – 1.44) | 0.37 | 83.0 | 93.6 | 1.13 (0.99 – 1.29) | 0.17 |
| 25-34 years | 56.0 | 77.0 | 1.37 (1.19 – 1.59) | <0.001* | 127.0 | 162.8 | 1.28 (1.16 – 1.41) | <0.001* |
| 35-44 years | 102.1 | 127.0 | 1.24 (1.11 – 1.40) | 0.001* | 162.6 | 206.1 | 1.27 (1.16 – 1.39) | <0.001* |
| 45-64 years | 385.6 | 419.6 | 1.09 (1.04 – 1.14) | 0.001* | 620.2 | 689.4 | 1.11 (1.07 – 1.15) | <0.001* |
| 65-84 years | 1986.3 | 2343.7 | 1.18 (1.15 – 1.21) | <0.001* | 2738.0 | 3198.1 | 1.17 (1.14 – 1.19) | <0.001* |
| ≥85 years | 14406.5 | 15790.2 | 1.10 (1.07 – 1.12) | <0.001* | 16409.7 | 18789.1 | 1.14 (1.11 – 1.18) | <0.001* |
| **Race** |  |  |  |  |  |  |  |  |
| Hispanic | 124.9 | 177.1 | 1.42 (1.22 – 1.65) | <0.001* | 180.5 | 295.8 | 1.64 (1.45 – 1.85) | <0.001* |
| Non-Hispanic American Indian | 889.8 | 1114.0 | 1.25 (1.09 – 1.43) | 0.005* | 994.2 | 1203.3 | 1.21 (1.06 – 1.38) | 0.01* |
| Non-Hispanic Asian/Pacific Islander | 230.7 | 321.2 | 1.39 (1.24 – 1.56) | <0.001* | 274.4 | 353.7 | 1.29 (1.16 – 1.44) | <0.001* |
| Non-Hispanic Black | 353.4 | 430.1 | 1.22 (1.12 – 1.33) | <0.001* | 436.3 | 610.8 | 1.40 (1.30 – 1.51) | <0.001* |
| Non-Hispanic White | 941.2 | 1050.2 | 1.12 (1.10 – 1.13) | <0.001* | 962.4 | 1088.4 | 1.13 (1.11 – 1.15) | <0.001* |
| **County of Residency** |  |  |  |  |  |  |  |  |
| Non-Rural Resident | 723.2 | 823.6 | 1.14 (1.12 – 1.16) | <0.001* | 744.1 | 868.3 | 1.17 (1.15 – 1.19) | <0.001* |
| Rural Resident | 1005.5 | 1109.5 | 1.10 (1.07 – 1.14) | <0.001* | 1064.9 | 1187.9 | 1.12 (1.08 – 1.15) | <0.001* |

**Supplemental Table 2: Minnesota Deaths by Discharge of Firearms, by Demographic Subgroup, 2018-2020.** Shown are the crude (unadjusted) mortality rates and rate ratios (RR) for each demographic subgroup in 2018-2019 and 2020. ^1^ P-values adjusted using multivariable Poisson regression accounting for age and sex. *Indicates statistical significance at p<0.05 after correcting for multiple comparisons using the Benjamini-Hochberg correction.

|  | **2018-2019** | | **2020** | | **Rate Ratio (RR)** | | | |
| --- | --- | --- | --- | --- | --- | --- | --- | --- |
| **Group** | **Deaths Total** | **Deaths Per 100,000** | **Deaths Total** | **Deaths Per 100,000** | **RR** | **95% CI** | **Unadj. P-Value** | **Adj. P-Value^1^** |
| **Age Group** |  |  |  |  |  |  |  |  |
| 0-4 years | 1 | 0.1 | 0 | 0.0 | — | — | — | — |
| 5-14 years | 2 | 0.1 | 1 | 0.1 | 0.99 | .09 - 10.87 | 1.00 | — |
| 15-24 years | 54 | 3.8 | 48 | 6.7 | 1.75 | 1.19 - 2.58 | 0.006* | 0.008* |
| 25-34 years | 53 | 3.4 | 49 | 6.2 | 1.82 | 1.24 - 2.69 | 0.003* | 0.01* |
| 35-44 years | 31 | 2.1 | 16 | 2.1 | 1.02 | 0.56 - 1.86 | 1.00 | 1.00 |
| 45-64 years | 18 | 0.6 | 21 | 1.5 | 2.30 | 1.23 - 4.32 | 0.01* | 0.03* |
| 65-84 years | 4 | 0.3 | 4 | 0.5 | 1.97 | 0.49 - 7.88 | 0.55 | — |
| ≥85 years | 0 | 0.0 | 0 | 0.0 | — | — | — | — |
| **Sex** |  |  |  |  |  |  |  |  |
| Male | 144 | 2.6 | 117 | 4.1 | 1.60 | 1.25 - 2.04 | <0.001* | <0.001* |
| Female | 19 | 0.3 | 22 | 0.8 | 2.28 | 1.24 - 4.22 | 0.01* | 0.02* |
| **Race** |  |  |  |  |  |  |  |  |
| Hispanic | 9 | 1.4 | 12 | 3.7 | 2.63 | 1.11 - 6.24 | 0.04* | 0.09 |
| Non-Hispanic American Indian | 16 | 13.0 | 7 | 11.2 | 0.86 | 0.36 - 2.10 | 0.92 | 0.97 |
| Non-Hispanic Asian/Pacific Islander | 3 | 0.5 | 2 | 0.6 | 1.31 | 0.22 - 7.86 | 1.00 | — |
| Non-Hispanic Black | 90 | 11.4 | 83 | 20.7 | 1.82 | 1.35 - 2.45 | <0.001* | <0.001* |
| Non-Hispanic White | 43 | 0.5 | 35 | 0.8 | 1.60 | 1.03 - 2.51 | 0.048* | 0.12 |
| **County of Residency** |  |  |  |  |  |  |  |  |
| Non-Rural Resident | 138 | 1.6 | 121 | 2.7 | 1.73 | 1.35 - 2.20 | <0.001* | <0.001* |
| Rural Resident | 25 | 1.0 | 18 | 1.5 | 1.42 | 0.78 - 2.61 | 0.33 | 0.44 |

**Supplemental Table 3: Minnesota Deaths by Discharge of Firearms, by Demographic Subgroup, Stratified by Sex, 2018-2020.** Shown are the crude (unadjusted) mortality rates and rate ratios (RR) for each demographic subgroup in 2018-2019 and 2020. *Indicates statistical significance at p<0.05 after correcting for multiple comparisons using the Benjamini-Hochberg correction.

|  | **Females** | | | | **Males** | | | |
| --- | --- | --- | --- | --- | --- | --- | --- | --- |
|  | **Deaths per 100,000** | | **RR**  **(95% CI)** | **P-Value** | **Deaths per 100,000** | | **RR**  **(95% CI)** | **P-Value** |
|  | **2018-2019** | **2020** |  |  | **2018-2019** | **2020** |  |  |
| **Age Group** |  |  |  |  |  |  |  |  |
| 0-4 years | 0.0 | 0.0 | --- | --- | 0.3 | 0.0 | --- | --- |
| 5-14 years | 0.0 | 0.0 | --- | --- | 0.3 | 0.3 | 0.99 (0.09 – 10.9) | >0.99 |
| 15-24 years | 0.3 | 1.1 | 3.94 (0.71 – 21.5) | 0.36 | 7.3 | 12.2 | 1.67 (1.12 – 2.49) | 0.04* |
| 25-34 years | 0.4 | 1.3 | 3.29 (0.78 – 13.7) | 0.33 | 6.3 | 10.9 | 1.73 (1.16 – 2.60) | 0.03* |
| 35-44 years | 1.0 | 1.3 | 1.41 (0.45 – 4.43) | 0.93 | 3.2 | 2.9 | 0.90 (0.44 – 1.84) | >0.99 |
| 45-64 years | 0.3 | 1.0 | 3.45 (1.01 – 11.8) | 0.16 | 1.0 | 2.0 | 1.97 (0.94 – 4.13) | 0.21 |
| 65-84 years | 0.4 | 0.2 | 0.66 (0.07 – 6.32) | >0.99 | 0.1 | 0.8 | 5.9 (0.62 – 56.9) | 0.38 |
| ≥85 years | 0.0 | 0.0 | --- |  | 0.0 | 0.0 | --- |  |
| **Race** |  |  |  |  |  |  |  |  |
| Hispanic | 0.3 | 0.0 | --- | >0.99 | 2.5 | 7.3 | 2.96 (1.21 – 7.23) | 0.06 |
| Non-Hispanic American Indian | 3.3 | 9.6 | 2.96 (0.49 – 17.7) | 0.65 | 22.7 | 12.8 | 0.56 (0.19 – 1.71) | 0.65 |
| Non-Hispanic Asian/Pacific Islander | 0.3 | 0.6 | 1.97 (0.12 – 31.5) | >0.99 | 0.7 | 0.7 | 0.98 (0.09 – 10.9) | >0.99 |
| Non-Hispanic Black | 1.0 | 3.5 | 3.50 (1.01 – 11.8) | 0.16 | 21.5 | 37.4 | 1.74 (1.28 – 2.37) | 0.002* |
| Non-Hispanic White | 0.3 | 0.5 | 1.97 (0.85 – 4.55) | 0.31 | 0.7 | 1.1 | 1.48 (0.87 – 2.51) | 0.34 |
| **County of Residency** |  |  |  |  |  |  |  |  |
| Non-Rural Resident | 0.3 | 0.9 | 3.03 (1.51 – 6.09) | 0.008* | 2.9 | 4.6 | 1.59 (1.22 – 2.07) | 0.003* |
| Rural Resident | 0.5 | 0.3 | 0.66 (0.13 – 3.26) | >0.99 | 1.6 | 2.6 | 1.66 (0.86 – 3.23) | 0.33 |

**Supplemental Table 4: Minnesota Deaths by Accidental Poisoning, by Demographic Subgroup, 2018-2020.** Shown are the crude (unadjusted) mortality rates and rate ratios (RR) for each demographic subgroup in 2018-2019 and 2020. ^1^ P-values adjusted using multivariable Poisson regression accounting for age and sex. *Indicates statistical significance at p<0.05 after correcting for multiple comparisons using the Benjamini-Hochberg correction.

|  | **2018-2019** | | **2020** | | **Rate Ratio (RR)** | | | |
| --- | --- | --- | --- | --- | --- | --- | --- | --- |
| **Group** | **Deaths Total** | **Deaths Per 100,000** | **Deaths Total** | **Deaths Per 100,000** | **RR** | **95% CI** | **Unadj. P-Value** | **Adj. P-Value^1^** |
| **Age Group** |  |  |  |  |  |  |  |  |
| 0-4 years | 0 | 0.0 | 3 | 0.8 | — | — | — | — |
| 5-14 years | 2 | 0.1 | 0 | 0.0 | — | — | — | — |
| 15-24 years | 141 | 10.0 | 117 | 16.3 | 1.64 | 1.28 - 2.09 | <0.001* | <0.001* |
| 25-34 years | 331 | 21.2 | 275 | 34.7 | 1.64 | 1.40 - 1.92 | <0.001* | <0.001* |
| 35-44 years | 257 | 17.4 | 204 | 27.2 | 1.56 | 1.30 - 1.88 | <0.001* | <0.001* |
| 45-64 years | 503 | 17.8 | 349 | 24.4 | 1.37 | 1.19 - 1.57 | <0.001* | <0.001* |
| 65-84 years | 69 | 4.4 | 39 | 4.9 | 1.11 | 0.75 - 1.65 | 0.66 | 0.59 |
| ≥85 years | 10 | 4.5 | 3 | 2.7 | 0.59 | 0.16 - 2.15 | 0.61 | 0.67 |
| **Sex** |  |  |  |  |  |  |  |  |
| Male | 889 | 16.0 | 681 | 24.1 | 1.51 | 1.37 - 1.67 | <0.001* | <0.001* |
| Female | 424 | 7.5 | 309 | 10.7 | 1.44 | 1.24 - 1.66 | <0.001* | <0.001* |
| **Race** |  |  |  |  |  |  |  |  |
| Hispanic | 52 | 8.1 | 45 | 13.8 | 1.71 | 1.14 - 2.54 | 0.01* | 0.02* |
| Non-Hispanic American Indian | 116 | 94.2 | 99 | 158.7 | 1.68 | 1.29 - 2.20 | <0.001* | <0.001* |
| Non-Hispanic Asian/Pacific Islander | 16 | 2.6 | 9 | 2.9 | 1.11 | 0.49 - 2.51 | 0.97 | 1.00 |
| Non-Hispanic Black | 201 | 25.4 | 176 | 43.8 | 1.72 | 1.41 - 2.11 | <0.001* | <0.001* |
| Non-Hispanic White | 921 | 10.6 | 657 | 14.9 | 1.41 | 1.27 - 1.55 | <0.001* | <0.001* |
| **County of Residency** |  |  |  |  |  |  |  |  |
| Non-Rural Resident | 1060 | 12.1 | 837 | 18.8 | 1.56 | 1.42 - 1.70 | <0.001* | <0.001* |
| Rural Resident | 253 | 10.3 | 153 | 12.3 | 1.19 | 0.98 - 1.46 | 0.09 | 0.17 |

**Supplemental Table 5: Minnesota Deaths by Accidental Poisoning, by Demographic Subgroup, Stratified by Sex, 2018-2020.** Shown are the crude (unadjusted) mortality rates and rate ratios (RR) for each demographic subgroup in 2018-2019 and 2020. *Indicates statistical significance at p<0.05 after correcting for multiple comparisons using the Benjamini-Hochberg correction.

|  | **Females** | | | | **Males** | | | |
| --- | --- | --- | --- | --- | --- | --- | --- | --- |
|  | **Deaths per 100,000** | | **RR**  **(95% CI)** | **P-Value** | **Deaths per 100,000** | | **RR**  **(95% CI)** | **P-Value** |
|  | **2018-2019** | **2020** |  |  | **2018-2019** | **2020** |  |  |
| **Age Group** |  |  |  |  |  |  |  |  |
| 0-4 years | 0.0 | 0.0 | --- | --- | 0.0 | 1.7 | --- | --- |
| 5-14 years | 0.3 | 0.0 | --- | --- | 0.0 | 0.0 | --- | --- |
| 15-24 years | 6.0 | 11.0 | 1.83 (1.18 – 2.83) | 0.03* | 13.9 | 21.5 | 1.55 (1.15 – 2.09) | 0.02* |
| 25-34 years | 11.9 | 18.2 | 1.54 (1.13 – 2.10) | 0.026 | 30.2 | 50.6 | 1.67 (1.39 – 2.02) | <0.001* |
| 35-44 years | 12.7 | 21.1 | 1.66 (1.23 – 2.23) | 0.005* | 22.0 | 33.2 | 1.51 (1.20 – 1.91) | 0.003* |
| 45-64 years | 11.6 | 14.5 | 1.25 (0.98 – 1.60) | 0.18 | 24.1 | 34.3 | 1.42 (1.21 – 1.68) | <0.001* |
| 65-84 years | 2.97 | 3.51 | 1.18 (0.62 – 2.24) | 0.90 | 5.9 | 6.4 | 1.08 (0.65 – 1.77) | >0.99 |
| ≥85 years | 4.20 | 1.38 | 0.33 (0.04 – 2.73) | 0.69 | 5.1 | 5.0 | 0.99 (0.18 – 5.38) | >0.99 |
| **Race** |  |  |  |  |  |  |  |  |
| Hispanic | 2.79 | 7.94 | 2.85 (1.22 – 6.66) | 0.06 | 13.3 | 19.6 | 1.47 (0.93 – 2.32) | 0.24 |
| Non-Hispanic American Indian | 100.9 | 173.4 | 1.72 (1.19 – 2.48) | 0.02* | 87.6 | 144.0 | 1.64 (1.11 – 2.44) | 0.05 |
| Non-Hispanic Asian/Pacific Islander | 1.56 | 1.84 | 1.18 (0.28 – 4.95) | >0.99 | 3.7 | 4.0 | 1.07 (0.40 – 2.91) | >0.99 |
| Non-Hispanic Black | 16.6 | 24.7 | 1.48 (1.02 – 2.15) | 0.11 | 34.0 | 62.5 | 1.84 (1.44 – 2.34) | <0.001* |
| Non-Hispanic White | 6.4 | 8.4 | 1.32 (1.10 – 1.59) | 0.01* | 14.9 | 21.5 | 1.44 (1.28 – 1.63) | <0.001* |
| **County of Residency** |  |  |  |  |  |  |  |  |
| Non-Rural Resident | 7.5 | 11.4 | 1.52 (1.30 – 1.79) | <0.001* | 16.7 | 26.3 | 1.57 (1.41 – 1.75) | <0.001* |
| Rural Resident | 7.4 | 8.3 | 1.12 (0.79 – 1.57) | 0.77 | 13.2 | 16.4 | 1.24 (0.97 – 1.59) | 0.21 |

**Supplemental Table 6: Minnesota Deaths by Malnutrition, by Demographic Subgroup, 2018-2020.** Shown are the crude (unadjusted) mortality rates and rate ratios (RR) for each demographic subgroup in 2018-2019 and 2020. ^1^ P-values adjusted using multivariable Poisson regression accounting for age and sex. *Indicates statistical significance at p<0.05 after correcting for multiple comparisons using the Benjamini-Hochberg correction.

|  | **2018-2019** | | **2020** | | **Rate Ratio (RR)** | | | |
| --- | --- | --- | --- | --- | --- | --- | --- | --- |
| **Group** | **Deaths Total** | **Deaths Per 100,000** | **Deaths Total** | **Deaths Per 100,000** | **RR** | **95% CI** | **Unadj. P-Value** | **Adj. P-Value^1^** |
| **Age Group** |  |  |  |  |  |  |  |  |
| 0-4 years | 0 | 0.0 | 0 | 0.0 | — | — | — | — |
| 5-14 years | 0 | 0.0 | 0 | 0.0 | — | — | — | — |
| 15-24 years | 1 | 0.1 | 0 | 0.0 | — | — | — | — |
| 25-34 years | 1 | 0.1 | 2 | 0.3 | 3.94 | 0.36 – 43.46 | 0.55 | — |
| 35-44 years | 2 | 0.1 | 0 | 0.0 | — | — | — | — |
| 45-64 years | 20 | 0.7 | 10 | 0.7 | 0.99 | 0.46 – 2.11 | 1.00 | 1.00 |
| 65-84 years | 64 | 4.0 | 43 | 5.4 | 1.32 | 0.90 – 1.95 | 0.18 | 0.30 |
| ≥85 years | 76 | 34.3 | 68 | 60.4 | 1.76 | 1.27 – 2.45 | <0.001* | 0.004* |
| **Sex** |  |  |  |  |  |  |  |  |
| Male | 62 | 1.1 | 38 | 1.3 | 1.21 | 0.81 – 1.81 | 0.42 | 0.54 |
| Female | 102 | 1.8 | 85 | 3.0 | 1.64 | 1.23 – 2.19 | <0.001* | 0.004* |
| **Race** |  |  |  |  |  |  |  |  |
| Hispanic | 0 | 0.0 | 2 | 0.6 | — | — | — | — |
| Non-Hispanic American Indian | 2 | 1.6 | 1 | 1.6 | 0.99 | 0.09 – 10.88 | 1.00 | — |
| Non-Hispanic Asian/Pacific Islander | 4 | 0.7 | 3 | 1.0 | 1.48 | 0.33 – 6.60 | 0.91 | — |
| Non-Hispanic Black | 2 | 0.3 | 3 | 0.8 | 2.95 | 0.49 – 17.68 | 0.44 | — |
| Non-Hispanic White | 156 | 1.8 | 114 | 2.6 | 1.44 | 1.13 – 1.83 | 0.004* | 0.007* |
| **County of Residency** |  |  |  |  |  |  |  |  |
| Non-Rural Resident | 134 | 1.5 | 85 | 1.9 | 1.25 | 0.95 – 1.64 | 0.12 | 0.11 |
| Rural Resident | 30 | 1.2 | 38 | 3.1 | 2.50 | 1.55 – 4.04 | <0.001* | 0.001* |

**Supplemental Table 7: Minnesota Deaths by Malnutrition, by Demographic Subgroup, Stratified by Sex, 2018-2020.** Shown are the crude (unadjusted) mortality rates and rate ratios (RR) for each demographic subgroup in 2018-2019 and 2020. *Indicates statistical significance at p<0.05 after correcting for multiple comparisons using the Benjamini-Hochberg correction.

|  | **Females** | | | | **Males** | | | |
| --- | --- | --- | --- | --- | --- | --- | --- | --- |
|  | **Deaths per 100,000** | | **RR**  **(95% CI)** | **P-Value** | **Deaths per 100,000** | | **RR**  **(95% CI)** | **P-Value** |
|  | **2018-2019** | **2020** |  |  | **2018-2019** | **2020** |  |  |
| **Age Group** |  |  |  |  |  |  |  |  |
| 0-4 years | 0.0 | 0.0 | --- | --- | 0.0 | 0.0 | --- | --- |
| 5-14 years | 0.0 | 0.0 | --- | --- | 0.0 | 0.0 | --- | --- |
| 15-24 years | 0.1 | 0.0 | --- | --- | 0.0 | 0.0 | --- | --- |
| 25-34 years | 0.0 | 0.5 | --- | --- | 0.1 | 0.0 | --- | --- |
| 35-44 years | 0.1 | 0.0 | --- | --- | 0.1 | 0.0 | --- | --- |
| 45-64 years | 0.8 | 1.1 | 1.43 (0.58 – 3.56) | 0.77 | 0.6 | 0.3 | 0.44 (0.09 – 2.03) | 0.65 |
| 65-84 years | 4.0 | 7.0 | 1.74 (1.06 – 2.84) | 0.09 | 4.1 | 3.5 | 0.85 (0.45 – 1.64) | 0.92 |
| ≥85 years | 38.5 | 62.2 | 1.61 (1.09 – 2.39) | 0.05 | 26.5 | 57.3 | 2.16 (1.20 – 3.90) | 0.04* |
| **Race** |  |  |  |  |  |  |  |  |
| Hispanic | 0.0 | 0.6 | --- | --- | 0.0 | 0.6 | --- | --- |
| Non-Hispanic American Indian | 3.3 | 3.2 | 0.99 (0.09 – 10.9) | >0.99 | 0.0 | 0.0 | --- | --- |
| Non-Hispanic Asian/Pacific Islander | 0.6 | 1.2 | 1.97 (0.28 – 14.0) | >0.99 | 0.7 | 0.7 | 0.98 (0.09 – 10.9) | >0.99 |
| Non-Hispanic Black | 0.3 | 1.0 | 3.94 (0.36 – 43.4) | 0.75 | 0.3 | 0.5 | 1.97 (0.12 – 31.5) | >0.99 |
| Non-Hispanic White | 2.2 | 3.5 | 1.61 (1.19 – 2.16) | 0.008* | 1.4 | 1.6 | 1.17 (0.77 – 1.78) | 0.74 |
| **County of Residency** |  |  |  |  |  |  |  |  |
| Non-Rural Resident | 1.9 | 2.5 | 1.34 (0.95 – 1.87) | 0.21 | 1.2 | 1.3 | 1.10 (0.69 – 1.75) | 0.93 |
| Rural Resident | 1.5 | 4.5 | 3.10 (1.70 – 5.56) | <0.001* | 1.0 | 1.6 | 1.65 (0.71 – 3.81) | 0.54 |

**Supplemental Table 8: Minnesota Deaths from Alcoholic Liver Disease, by Demographic Subgroup, 2018-2020.** Shown are the crude (unadjusted) mortality rates and rate ratios (RR) for each demographic subgroup in 2018-2019 and 2020. ^1^ P-values adjusted using multivariable Poisson regression accounting for age and sex. *Indicates statistical significance at p<0.05 after correcting for multiple comparisons using the Benjamini-Hochberg correction.

|  | **2018-2019** | | **2020** | | **Rate Ratio (RR)** | | | |
| --- | --- | --- | --- | --- | --- | --- | --- | --- |
| **Group** | **Deaths Total** | **Deaths Per 100,000** | **Deaths Total** | **Deaths Per 100,000** | **RR** | **95% CI** | **Unadj. P-Value** | **Adj. P-Value^1^** |
| **Age Group** |  |  |  |  |  |  |  |  |
| 0-4 years | 0 | 0.0 | 0 | 0.0 | — | — | — | — |
| 5-14 years | 0 | 0.0 | 0 | 0.0 | — | — | — | — |
| 15-24 years | 0 | 0.0 | 0 | 0.0 | — | — | — | — |
| 25-34 years | 36 | 2.3 | 39 | 4.9 | 2.13 | 1.36 - 3.36 | 0.001* | 0.004* |
| 35-44 years | 111 | 7.5 | 85 | 11.3 | 1.51 | 1.14 - 2.00 | 0.005* | 0.01* |
| 45-64 years | 567 | 20.1 | 335 | 23.4 | 1.16 | 1.02 - 1.33 | 0.03* | 0.05 |
| 65-84 years | 248 | 15.7 | 150 | 18.7 | 1.19 | 0.97 - 1.46 | 0.10 | 0.13 |
| ≥85 years | 1 | 0.5 | 8 | 7.1 | 15.77 | 1.97 - 126.10 | 0.002* | 0.02* |
| **Sex** |  |  |  |  |  |  |  |  |
| Male | 617 | 11.1 | 372 | 13.2 | 1.19 | 1.04 - 1.35 | 0.009* | 0.02* |
| Female | 346 | 6.1 | 245 | 8.5 | 1.40 | 1.18 - 1.64 | <0.001* | <0.001* |
| **Race** |  |  |  |  |  |  |  |  |
| Hispanic | 22 | 3.4 | 16 | 4.9 | 1.43 | 0.75 - 2.73 | 0.35 | 0.49 |
| Non-Hispanic American Indian | 65 | 52.8 | 45 | 72.1 | 1.37 | 0.93 - 2.00 | 0.13 | 0.27 |
| Non-Hispanic Asian/Pacific Islander | 11 | 1.8 | 10 | 3.2 | 1.79 | 0.76 - 4.22 | 0.26 | 0.38 |
| Non-Hispanic Black | 38 | 4.8 | 22 | 5.5 | 1.14 | 0.67 - 1.93 | 0.72 | 0.85 |
| Non-Hispanic White | 821 | 9.4 | 523 | 11.8 | 1.26 | 1.13 - 1.40 | <0.001* | <0.001* |
| **County of Residency** |  |  |  |  |  |  |  |  |
| Non-Rural Resident | 758 | 8.6 | 495 | 11.1 | 1.29 | 1.15 - 1.44 | <0.001* | <0.001* |
| Rural Resident | 205 | 8.3 | 122 | 9.8 | 1.18 | 0.94 - 1.47 | 0.17 | 0.34 |

**Supplemental Table 9: Minnesota Deaths from Alcoholic Liver Disease, by Demographic Subgroup, Stratified by Sex, 2018-2020.** Shown are the crude (unadjusted) mortality rates and rate ratios (RR) for each demographic subgroup in 2018-2019 and 2020. *Indicates statistical significance at p<0.05 after correcting for multiple comparisons using the Benjamini-Hochberg correction.

|  | **Females** | | | | **Males** | | | |
| --- | --- | --- | --- | --- | --- | --- | --- | --- |
|  | **Deaths per 100,000** | | **RR**  **(95% CI)** | **P-Value** | **Deaths per 100,000** | | **RR**  **(95% CI)** | **P-Value** |
|  | **2018-2019** | **2020** |  |  | **2018-2019** | **2020** |  |  |
| **Age Group** |  |  |  |  |  |  |  |  |
| 0-4 years | 0.0 | 0.0 | --- | --- | 0.0 | 0.0 | --- | --- |
| 5-14 years | 0.0 | 0.0 | --- | --- | 0.0 | 0.0 | --- | --- |
| 15-24 years | 0.0 | 0.0 | --- | --- | 0.0 | 0.0 | --- | --- |
| 25-34 years | 2.5 | 5.7 | 2.28 (1.23 – 4.22) | 0.03* | 2.1 | 4.2 | 1.97 (1.01 – 3.86) | 0.15 |
| 35-44 years | 6.8 | 10.4 | 1.54 (1.01 – 2.34) | 0.13 | 8.2 | 12.2 | 1.49 (1.01 – 2.18) | 0.13 |
| 45-64 years | 14.5 | 18.7 | 1.29 (1.04 – 1.60) | 0.07 | 25.7 | 28.1 | 1.09 (0.92 – 1.30) | 0.52 |
| 65-84 years | 8.5 | 11.5 | 1.34 (0.93 – 1.93) | 0.26 | 23.8 | 26.9 | 1.13 (0.89 – 1.44) | 0.55 |
| ≥85 years | 0.0 | 1.4 | --- | 0.90 | 1.3 | 17.4 | 13.80 (1.70 – 112.2) | 0.02* |
| **Race** |  |  |  |  |  |  |  |  |
| Hispanic | 1.2 | 2.4 | 1.97 (0.49 – 7.88) | 0.75 | 5.6 | 7.3 | 1.31 (0.63 – 2.73) | 0.77 |
| Non-Hispanic American Indian | 65.1 | 83.5 | 1.28 (0.78 – 2.10) | 0.59 | 40.6 | 60.8 | 1.50 (0.83 – 2.72) | 0.39 |
| Non-Hispanic Asian/Pacific Islander | 1.9 | 2.5 | 1.31 (0.37 – 4.65) | >0.99 | 1.7 | 4.0 | 2.36 (0.72 – 7.74) | 0.41 |
| Non-Hispanic Black | 3.3 | 6.6 | 1.97 (0.91 – 4.25) | 0.23 | 6.2 | 4.4 | 0.71 (0.33 – 1.52) | 0.68 |
| Non-Hispanic White | 6.4 | 8.8 | 1.38 (1.15 – 1.66) | 0.003* | 12.5 | 14.9 | 1.19 (1.04 – 1.37) | 0.04* |
| **County of Residency** |  |  |  |  |  |  |  |  |
| Non-Rural Resident | 6.2 | 8.9 | 1.42 (1.19 – 1.71) | <0.001* | 11.1 | 13.4 | 1.21 (1.04 – 1.40) | 0.04* |
| Rural Resident | 5.6 | 7.2 | 1.29 (0.88 – 1.88) | 0.38 | 11.2 | 12.5 | 1.12 (0.85 – 1.48) | 0.68 |

**Supplemental Table 10: Minnesota Deaths from Cirrhosis or Other Chronic Liver Disease, by Demographic Subgroup, 2018-2020.** Shown are the crude (unadjusted) mortality rates and rate ratios (RR) for each demographic subgroup in 2018-2019 and 2020. ^1^ P-values adjusted using multivariable Poisson regression accounting for age and sex. *Indicates statistical significance at p<0.05 after correcting for multiple comparisons using the Benjamini-Hochberg correction.

|  | **2018-2019** | | **2020** | | **Rate Ratio (RR)** | | | |
| --- | --- | --- | --- | --- | --- | --- | --- | --- |
| **Group** | **Deaths Total** | **Deaths Per 100,000** | **Deaths Total** | **Deaths Per 100,000** | **RR** | **95% CI** | **Unadj. P-Value** | **Adj. P-Value^1^** |
| **Age Group** |  |  |  |  |  |  |  |  |
| 0-4 years | 0 | 0.0 | 0 | 0.0 | — | — | — | — |
| 5-14 years | 0 | 0.0 | 0 | 0.0 | — | — | — | — |
| 15-24 years | 0 | 0.0 | 0 | 0.0 | — | — | — | — |
| 25-34 years | 6 | 0.4 | 8 | 1.0 | 2.63 | 0.91 - 7.57 | 0.12 | 0.17 |
| 35-44 years | 13 | 0.9 | 10 | 1.3 | 1.52 | 0.66 - 3.46 | 0.44 | 0.56 |
| 45-64 years | 132 | 4.7 | 86 | 6.0 | 1.28 | 0.98 - 1.68 | 0.08 | 0.18 |
| 65-84 years | 210 | 13.3 | 141 | 17.6 | 1.32 | 1.07 - 1.64 | 0.01* | 0.04* |
| ≥85 years | 41 | 18.5 | 16 | 14.2 | 0.77 | 0.43 - 1.37 | 0.45 | 0.59 |
| **Sex** |  |  |  |  |  |  |  |  |
| Male | 209 | 3.8 | 149 | 5.3 | 1.40 | 1.14 - 1.73 | 0.002* | 0.008* |
| Female | 193 | 3.4 | 112 | 3.9 | 1.14 | 0.91 - 1.44 | 0.28 | 0.44 |
| **Race** |  |  |  |  |  |  |  |  |
| Hispanic | 10 | 1.6 | 7 | 2.1 | 1.38 | 0.53 - 3.62 | 0.69 | 0.51 |
| Non-Hispanic American Indian | 14 | 11.4 | 6 | 9.6 | 0.85 | 0.33 - 2.20 | 0.91 | 0.94 |
| Non-Hispanic Asian/Pacific Islander | 11 | 1.8 | 6 | 1.9 | 1.07 | 0.40 - 2.91 | 1.00 | 1.00 |
| Non-Hispanic Black | 17 | 2.2 | 10 | 2.5 | 1.16 | 0.53 - 2.53 | 0.87 | 0.95 |
| Non-Hispanic White | 348 | 4.0 | 232 | 5.3 | 1.31 | 1.11 - 1.55 | 0.001* | 0.006* |
| **County of Residency** |  |  |  |  |  |  |  |  |
| Non-Rural Resident | 293 | 3.3 | 192 | 4.3 | 1.29 | 1.08 - 1.55 | 0.007* | 0.02* |
| Rural Resident | 109 | 4.4 | 69 | 5.5 | 1.25 | 0.93 - 1.69 | 0.17 | 0.34 |

**Supplemental Table 11: Minnesota Deaths from Cirrhosis or Other Chronic Liver Disease, by Demographic Subgroup, Stratified by Sex, 2018-2020.** Shown are the crude (unadjusted) mortality rates and rate ratios (RR) for each demographic subgroup in 2018-2019 and 2020. *Indicates statistical significance at p<0.05 after correcting for multiple comparisons using the Benjamini-Hochberg correction.

|  | **Females** | | | | **Males** | | | |
| --- | --- | --- | --- | --- | --- | --- | --- | --- |
|  | **Deaths per 100,000** | | **RR**  **(95% CI)** | **P-Value** | **Deaths per 100,000** | | **RR**  **(95% CI)** | **P-Value** |
|  | **2018-2019** | **2020** |  |  | **2018-2019** | **2020** |  |  |
| **Age Group** |  |  |  |  |  |  |  |  |
| 0-4 years | 0.0 | 0.0 | --- | --- | 0.0 | 0.0 | --- | --- |
| 5-14 years | 0.0 | 0.0 | --- | --- | 0.0 | 0.0 | --- | --- |
| 15-24 years | 0.0 | 0.0 | --- | --- | 0.0 | 0.0 | --- | --- |
| 25-34 years | 0.1 | 0.3 | 1.97 (0.12 – 31.5) | >0.99 | 0.6 | 1.7 | 2.76 (0.88 – 8.69) | 0.25 |
| 35-44 years | 0.8 | 1.3 | 1.64 (0.50 – 5.38) | 0.78 | 0.9 | 1.3 | 1.41 (0.45 – 4.43) | 0.94 |
| 45-64 years | 4.2 | 5.0 | 1.20 (0.79 – 1.82) | 0.65 | 5.2 | 7.0 | 1.35 (0.94 – 1.93) | 0.24 |
| 65-84 years | 12.8 | 14.3 | 1.11 (0.81 – 1.52) | 0.75 | 13.8 | 21.3 | 1.55 (1.15 – 2.07) | 0.02* |
| ≥85 years | 13.3 | 12.4 | 0.93 (0.42 – 2.06) | >0.99 | 27.8 | 17.4 | 0.63 (0.27 – 1.47) | 0.58 |
| **Race** |  |  |  |  |  |  |  |  |
| Hispanic | 0.9 | 0.6 | 0.66 (0.07 – 6.32) | >0.99 | 2.2 | 3.7 | 1.69 (0.57 – 5.03) | 0.73 |
| Non-Hispanic American Indian | 16.3 | 9.6 | 0.59 (0.16 – 2.15) | 0.78 | 6.5 | 9.6 | 1.48 (0.33 – 6.61) | >0.99 |
| Non-Hispanic Asian/Pacific Islander | 2.2 | 1.2 | 0.56 (0.12 – 2.71) | 0.90 | 1.3 | 2.6 | 1.97 (0.49 – 7.88) | 0.76 |
| Non-Hispanic Black | 2.3 | 0.5 | 0.22 (0.03 – 1.73) | 0.37 | 2.0 | 4.4 | 2.22 (0.85 – 5.74) | 0.29 |
| Non-Hispanic White | 3.7 | 4.7 | 1.26 (0.99 – 1.61) | 0.16 | 4.3 | 5.8 | 1.36 (1.09 – 1.71) | 0.03* |
| **County of Residency** |  |  |  |  |  |  |  |  |
| Non-Rural Resident | 3.1 | 3.9 | 1.27 (0.97 – 1.65) | 0.20 | 3.6 | 4.7 | 1.31 (1.02 – 1.68) | 0.09 |
| Rural Resident | 4.5 | 3.8 | 0.85 (0.52 – 1.37) | 0.77 | 4.3 | 7.3 | 1.68 (1.13 – 2.50) | 0.04* |
